# Construction and evaluation of animal models of endplate injury: a systematic review

**DOI:** 10.1186/s12891-026-09652-w

**Published:** 2026-02-25

**Authors:** Ningning Feng, Shuyin Tan, Xing Yu, Yukun Ma, Ziye Qiu, Sixue Chen, Luchun Xu, Guozheng Jiang, Jiawei Song, Wenhao Li

**Affiliations:** 1 Dongzhimen Hospital Affiliated to Beijing University of Chinese Medicine, Beijing, 100700 China; 2 Beijing Hospital of Traditional Chinese Medicine Affiliated to Capital Medical University, Beijing, 100010 China

**Keywords:** Endplate injury, Modic changes, Animal models, Systematic review

## Abstract

**Purpose:**

A systematic review of animal models for endplate injury was conducted to identify an appropriate model for investigating the pathophysiological mechanisms underlying endplate injury and its association with low back pain (LBP).

**Methods:**

A comprehensive search of relevant literature was conducted using electronic databases. The identified studies were then evaluated based on predefined inclusion and exclusion criteria. Subsequently, key information and primary experimental findings from the selected literature were extracted and synthesized. This research was performed in accordance with the guidelines outlined in the Preferred Reporting Items for Systematic Reviews and Meta-Analyses (PRISMA).

**Results:**

Our study incorporated a total of 10 papers, covering a span of 8 years. Among these, 2 studies specifically induced injury to the endplate and intervertebral disc, respectively. 6 studies involved puncturing the endplate or intervertebral disc followed by the injection of various substances. Additionally, 1 study focused on modeling lumbar instability, while another modeled the tamponade of the nucleus pulposus within the vertebral body. The subjects primarily consisted of mice and rabbits, with 2 studies utilizing mice and 5 studies selecting rats. The remaining 3 studies employed New Zealand White rabbits. The observation period for the experimental models ranged from 4 to 24 weeks postoperatively. The assessment of the animal models predominantly included imaging, behavioral evaluations, histological analyses, and molecular biological tests, with MRI and histological examination being the most frequently utilized methods.

**Conclusions:**

The research has found that endplate Modic changes and endplate injuries are closely related to LBP. Animal experiments provide a model reference for exploring the intrinsic mechanism between the two and the corresponding therapeutic options. Although different modeling methods all induce varying degrees of endplate damage, each has its own advantages and disadvantages. The occurrence process of diseases in animal models is not completely equivalent to that in the human body, and there is currently no unified standard for clinical judgment of such models. Therefore, when choosing an animal model, multiple considerations should be integrated.

**Supplementary Information:**

The online version contains supplementary material available at 10.1186/s12891-026-09652-w.

## Introduction

Low back pain (LBP) is the most prevalent musculoskeletal disorder in clinical practice, significantly impairing patients’ quality of life and imposing substantial socioeconomic burdens [1, 2]. LBP is particularly common in developed countries. Reports indicate that up to 25% of Americans experienced LBP within the past three months [3]. The annual prevalence of LBP in the general population is 38%, with a lifetime prevalence reaching 40% [4]. The pathogenesis of LBP is relatively complex, and previous research has primarily focused on the association between intervertebral disc degeneration and LBP [5]. Modic Changes refer to MRI signal alterations in the vertebral body bone marrow near the endplates [6, 7]. With the widespread clinical application of MRI, increasing attention has been drawn to their association with low back pain. Studies have shown that the prevalence of MCs in individuals with LBP is seven times higher than in the general population, establishing MCs as a risk factor for LBP [8–10].

The vertebral endplate comprises both a bony endplate and a cartilaginous endplate. The bony endplate, formed by the ossification of the epiphyseal plate on the superior and inferior surfaces of the vertebral body, exhibits a slight concavity. The cartilaginous endplate consists of a thin central layer of hyaline cartilage. Research has revealed [11] that endplate innervation originates primarily from the sinuvertebral nerve. After branching from the ventral ramus and gray ramus communicans of the spinal nerve, the sinuvertebral nerve courses along the posterior longitudinal ligament and typically divides into three branches, innervating the posterior aspects of the intervertebral disc and vertebral body. In the central region of the posterior vertebral cortex, the basivertebral nerve (BVN), a branch of the sinuvertebral nerve, accompanies the basivertebral vessels through the basivertebral foramen into the vertebral body center, forming a neurovascular complex. Branches from this complex extend uniformly to the superior and inferior vertebral bodies, innervating the bony endplates. In endplate pathology or disc degeneration, sensory and sympathetic innervation of the vertebral endplate increases, accompanied by the expression of pain markers such as substance P [12, 13]. Additionally, intraosseous basivertebral nerve radiofrequency ablation (IBNRA) can induce degeneration of the vertebral nerve in the affected vertebrae through targeted thermal ablation. This intervention can interrupt the nociceptive signaling from the basivertebral nerve, thereby alleviating low back pain associated with endplate lesions [14, 15]. These findings further suggest that endplate microfractures or lesions may represent a significant source of lower back pain [16–18].

The pathophysiologic mechanisms of endplate injury are not fully understood. Only few studies have focused on endplate injury and the construction of animal models of endplate injury. In this paper, we systematically review the current animal models regarding lumbar endplate lesions to provide an animal model reference for exploring the intrinsic mechanism between endplate degeneration or endplate injury and LBP and the corresponding therapeutic options.

## Methods

This study was conducted according to the statements in the Preferred Reporting Items for Systems Reviews and Meta-Analyses (PRISMA) [19]. Because this study was a review of published research, it did not require ethical approval.

### Literature search

We use the terms “endplate injury”, “endplate lesion”,“endplate damage ”, “endplate modic changes”, “modic changes”,“animal models ”, “experimental animal”, and their combined forms were electronically searched in the following databases: PubMed, Cochrane Library, Electronic Databases, Scientific Databases, China Biomedical Database (CBM), China Wikipedia, China National Knowledge Infrastructure (CNKI), and Wanfang.

### Inclusion and exclusion criteria

Inclusion criteria：1. Object of study: the animal model used to study end-plate injury in vivo is not limited to species, gender, age, etc. 2. Main outcome indicators: the model animals had typical imaging or histological findings related to end-plate injury, and relevant experimental data were included. 3. Research type: related research on animal experiments. 4. Language type: unlimited.

Exclusion criteria: 1. The molding method and effect are not clearly described; 2. The subjects are not animals; 3. No molding or in vitro molding; 4. The types of articles are review, systematic review, etc. For studies that met the inclusion criteria, we also traced their references to identify potential target literature. Literature search and screening was carried out independently by two researchers. Any disagreements that arose during the process were resolved through discussion and agreement was reached.

### Data extraction

According to the purpose of our study, we extracted the following information from the included literature: the first author of the literature, the year of publication, the type of animal, the location and segment of the modeling access, the specific modeling method, the time of observation of the model, and the model evaluation indexes and results.

### Literature quality assessment

We used the initial Stroke Therapy Academic Industry Roundtable list (STAIR) to evaluate the quality of the included animal experiments.

## Results

### Results of the literature search

**Fig. 1:**
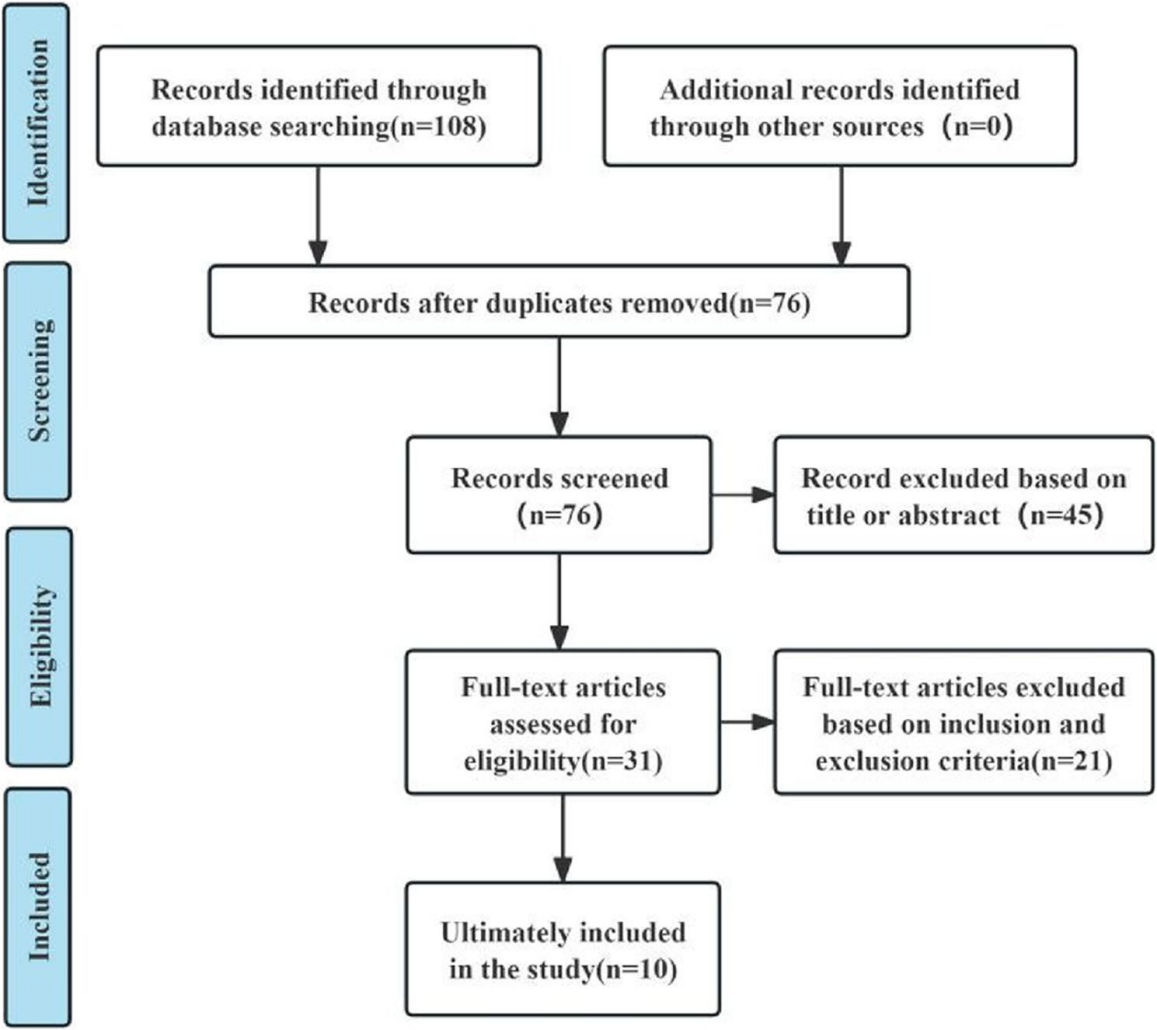
Flowchart of literature inclusion

Based on the search strategy implemented, a total of 108 pertinent documents were identified, with 76 remaining after the removal of duplicates. Following a review of titles and abstracts, 45 studies were excluded. The remaining 31 articles underwent full-text assessment, resulting in the exclusion of 21 studies that did not satisfy the inclusion criteria. Ultimately, 10 studies were incorporated into this analysis, comprising 1 Chinese-language article and 9 English-language articles. The literature screening process is illustrated in Fig. 1.

### Results of animal experiments

The 10 literatures included were mainly from the United States, China and Japan. The earliest study was published in 2016 [20–22], and the latest one was published in 2024 [23]. In terms of selection of experimental animals: Among the 10 studies, 7 studies selected mice as experimental objects [22, 23, 25–29], among which 5 studies selected rats [22, 23, 27–29], and 2 studies selected male mice [25, 26]. Three other studies selected male New Zealand white rabbits as subjects [20, 21, 24]. Nine studies reported the weekly age of experimental animals. Of the seven studies with rats as the research object, three reported the weekly age of rats was 8w [22, 26, 27], two reported the weekly age of rats was 12w [23, 25], and the other two reported the weekly age of rats was 20w or above [28, 29]. Among the two studies that took New Zealand white rabbits as the research object, one study reported that the rabbits were 12w old [24], and the other study reported that the rabbits were 12 to 14w old [20].

Selection of surgical approach: 4 studies selected abdominal approach [23, 27–29], 2 studies selected posterolateral peritoneal approach [20, 21], 2 studies selected caudal approach [22, 25], and 2 studies selected dorsal approach [24, 26]. Selection of surgical segments: 6 studies selected different disk segments of L3-L6 as surgical operation locations [21, 23, 24, 26–28], 2 studies selected L4, L4 and L5 vertebrae for surgical operations [20, 29], and the other 2 studies selected different disk segments of Ca2-8 for surgical operations [22, 25].

Establishment of endplate injury models: 2 studies directly damaged the disc or vertebral body [25, 27], 6 studies injected different substances after endplate or disc puncture [21–24, 28, 29], 1 study established the lumbar instability model [26], and 1 study implanted the nucleus pulposus tissue in the vertebral body [20]. The observation period of the included studies ranged from 4w to 24w after surgery. The postoperative samples mainly included lumbar vertebra, tail vertebra, intervertebral disc, dorsal root ganglion and spinal cord tissue.

The evaluation indexes of the model mainly included imaging, behavioral, histological and molecular biological examinations. Among the 10 studies, MRI was used in 6 studies [20–22, 24, 27, 28] and µCT was used in 5 studies [23, 24, 26–28]. 5 studies observed behavioral changes in rats. Histologic examination was performed in all studies, and molecular biology was also performed in three studies [20, 24, 26]. The basic characteristics of the included literatures are shown in Table1, and the model evaluation indicators and results are shown in Table 2.

**Table 1:** Different animal models of endplate injury

Author	Country	Week old	Animal type	Operation method	Observation time
(1)Nucleus pulposus implantation model					
Han et al[20], 2016	China	12-14w	Male New Zealand white rabbit	ME group (*n* = 18)/NPE group (*n* = 18): The right anterolateral spine from L1 to L6 was exposed and the L5 vertebra adjacent to the endplate was drilled with a 16G needle to a depth of 3 mm. Autologous NPs were obtained by aspiration in the L1-L2 intervertebral disc using a 5 ml syringe. The muscle or nucleus pulposus tissue is transplanted into the borehole and the incisions are closed layer by layer.	Postoperative 12, 16, 20w
				Sham surgery group (*n* = 18): Drill and fill the L5 vertebrae as described above without inserting anything, closing the incision layer by layer.	
(2)Mechanical damage model					
Zuo R[25], 2020	China	12w	Male C57 mice	Model group (*n* = 10): after anesthesia and disinfection, rubber bands were ligated proximal to the tail of mice. An incision was made on the left side of the h tail, about 1.5 cm long, to reveal the mouse intervertebral disc, and the caudal vertebrae were cut at 1 mm below the endplates of vertebrae 6, 7 and 8 of the mouse tail body near the disc.	Postoperative 4w
				Sham operation group (*n* = 10): just follow the above steps to reveal the disc.	
Morisako et al[27], 2022	Japan	8w	Female SD rats	EPL group: a 2 × 4 mm surgical incision was created in the anterior fibrous ring with a scalpel, and the medullary tissue was scraped out with a micro-scraper, preserving the bilateral and posterior fibrous rings and cartilaginous endplates.	Postoperative 1, 4, 8, 12w
				Sham-operation group: same surgical approach with exposure of L4−5 disc only.	
Wang et al[28], 2023	USA	20w	Male SD rats	EP + PBS(*n* = 6)/EP + TNF-α(*n* = 7) group: cephalad EP of L4–5 and L5–6 IVDs was punctured obliquely from 1.5 mm cephalad of the vertebral body using a 0.6 mm Gram needle to a depth of 3 mm. 2.5 ul of PBS or TNF-α was injected into the discs after the EP puncture injury, respectively.	Postoperative 8w
				Sham-operation group (*n* = 6): only the vertebral bodies and intervertebral discs between L4 and L6 were exposed.	
Lai et al[29], 2023	USA	20−24w	Male SD rats	EP + PBS(*n* = 5)/EP + TNF-α(*n* = 5) group: cephalad EP of L4–5 and L5–6 IVDs were punctured obliquely from 1.5 mm cephalad of the vertebral body using a 0.6 mm Gram needle to a depth of 3 mm. 2.5 ul of PBS or TNF-α was injected into the discs after the EP puncture injury, respectively.	Postoperative 8w
				Sham-operation group (*n* = 5): only the vertebral bodies and intervertebral discs between L4 and L6 were exposed.	
(3)Bacterial infection model					
Chen et al[2^1^], 2016	China	-	New Zealand white rabbit	A group (*n* = 3):After anesthesia, the L5-L7 vertebral bodies and intervertebral discs were exposed layer by layer, and the nucleus pulposus was punctured to a depth of 5–6 mm using a syringe with a 28G needle.	Postoperative 2, 8 w
				B group (*n* = 2): The same steps were performed as described above, with the difference that the L6−7 interspaces were punctured and inoculated with 25 µl of the standard strain of Propionibacterium acnes.	
				C group (*n* = 3): The same steps were performed as described above, with the difference that interspaces L5−6 were punctured and inoculated with 25 µl of saline, and interspaces L6−7 were punctured and inoculated with 25 µl of Staphylococcus aureus.	
Dudli et al[22], 2016	USA	8w	Female SD rats	Model group (*n* = 10): after anesthesia, Ca2−8 was exposed layer by layer, and 1.5 µl of Propionibacterium acnes was injected into the Ca2−3, Ca4−6, and Ca7−8 interstitial discs by puncturing and aspirating a portion of the medullary tissue of Ca2−8 using a syringe with a 28G needle.	Postoperative 1, 3, 7, 14, 20 d
				Control group (*n* = 10): same steps as above, but 1.5 µl PBS was disked between Ca3−4 and Ca6−7.	
Shan et al[24], 2017	China	12w	Male New Zealand Rabbit	Model group (*n* = 10): 1 ml of Propionibacterium acnes was slowly injected into the marrow cavity under the cartilaginous endplates using a 22G needle screwed into the cortical bone from the right side of the rat into the subchondral bone above the L4-L5 and L5-L6 discs, with the tip of the needle 1–2 mm away from the adjacent endplates at an angle of 45° to the coronal plane and the cross-section.	Postoperative 2, 4, 8, 12, 24w
				Control group (*n* = 10): same procedure as above, but 1 ml saline was chosen as injection solution.	
(4)Chemical-induced metabolic damage model					
Maruyama et al[23],2024	Japan	12w	Female SD rats	Model group (*n* = 72): The anterior portions of the L3−4, L4−5, and L5−6 intervertebral discs were exposed via a transabdominal approach. A 27-gauge syringe was used to inject 0.5 mg/1 mg MIA into the L4−5 and L5−6 intervertebral discs, respectively.	Postoperative 3, 6, 12w
				Control group (*n* = 72): same steps as above, but only the L3−4 disc was exposed without injecting anything.	
(5)Lumbar instability model					
Liu et al[26], 2021	Chian	8w	Male C57 mice	LSI group: After anesthesia, the paravertebral muscles were isolated layer by layer, the L3−5 spinous processes of mice were exposed, and the supraspinous and interspinous ligaments of the L3-L5 vertebrae were excised to create a model of the LSI that leads to vertebral endplate degeneration.	Postoperative 4, 8w
				Sham operation group: only the paravertebral muscles of L3−5 were isolated.	

*NP* nucleus pulposus, *W* Weeks, * L* Lumbar, *D* day, * Ca* caudal vertebrae, *EP* endplate, *MIA* monosodium iodoacetate

**Table 2:** Evaluation indexes for animal models of endplate injury

Author	Imaging results		Pain behavior results	Histological results	Immunohistochemistry/ fluorescence results	RT-qPCR/WB Results
	MRI	µCT				
(1)Nucleus pulposus implantation model						
Han et al. [20],2016	In the NPE group, the L5 vertebrae showed low signal in the T1-weighted image and medium-low signal changes in the T2-weighted image at different postoperative time points.	-	-	HE results showed that chondrocytes and myeloid cells in the NPE group were significantly increased in value, with different cell shapes and sizes.	-	RT-qPCR results showed that the mRNA expression of IL−4, IL−17 and IFN-γ was significantly higher in the NPE group compared with the ME group and the sham-operated group.WB results showed that the levels of IL−4 and IL−17 proteins in the NPE group were significantly higher compared with the ME group and the sham-operated group.
(2)Mechanical damage model						
Zuo R[25], 2020	-	-	-	Safranin O and Fast Green staining revealed that rats in the model group had a significant decrease in senna staining of the cartilage endplate at the fracture site and a large amount of inflammatory tissue proliferation around the fracture.	Immunohistochemical results showed that rats in the model group had a large amount of tissue with high expression of TNF-α and inflammatory cell infiltration in the fracture region.	-
Morisako et al[27],2022	In the EPL group, low-signal changes in the vertebral endplate region were observed on MRI T1- and T2-weighted images at 8 and 12 w postoperatively.	At 12w after operation, the BV/TV value of EPL group was significantly higher than that of sham group.	At 12w after operation, the hair stroking time of EPL group increased significantly, and the exercise and standing time decreased.	At 12w after surgery, safranin O and Fast Green staining in the EPL group showed that the vertebral endplate was highly degraded and the trabecular bone in the vertebral body was irregular.	At 12w after operation, the number of TRAP-positive cells in EPL group was significantly higher than that in sham operation group. The results of CGRP immunostaining showed that the percentage of CGRP positive area around the endplate in EPL group was significantly higher than that in sham operation group.	-
Wang et al[28], 2023	Modic I-type changes were seen in both EP injury groups, with MRI T1-weighted images showing low signal and T2-weighted images showing high signal.	µCT showed that endplate structure was disrupted and bone trabeculae were remodeled in the rats of the EP injury group.	The hind paw retraction threshold and forepaw grip strength were significantly decreased in the EP-injured group of rats.	Safranin O and Fast Green staining showed that rats in the endplate injury group underwent moderate-to-severe mesenchymal degeneration.	Immunofluorescence results showed that the percentage of substance P in the dorsal horn of the spinal cord of rats in the EP-injured group was significantly increased compared with that in the sham-operated group.	-
Lai et al[29], 2023	-	-	The hind paw retraction threshold and forepaw grip strength were significantly decreased in the EP-injured group of rats.	Safranin O and Fast Green staining showed that the height of the intervertebral disc was reduced in the endplate injury group of rats.	Immunofluorescence results showed that the percentages of substance P, Iba1-ir and GFAP-ir in the dorsal horn of the spinal cord of rats in the EP-injured group were significantly increased. Toluidine blue CD68 immunohistochemical staining showed that the EP structure was disrupted in the model group, and CD68-ir was strongly expressed in the vertebral and cartilage EP regions close to the injury site.	-
(3)Bacterial infection model						
Chen et al. [21],2016	At 2−8w after surgery, MRI showed changes in end-plate signal intensity at L6−7 segments of rabbits in both A/B groups, with low signal intensity in T1W1 and high signal intensity in T2W1.	-	-	Endplate rupture and disorganization of the nucleus pulposus and annulus fibrosus were observed in segments inoculated with Propionibacterium acnes in Groups A/B.	-	-
Dudli et al[22], 2016	The 20d postoperative MRI results showed signal changes in the endplate of the Propionibacterium acnes-injected segment, which appeared as low or equal signal in T1W1 and high signal changes in T2W1.	-	-	Heidenhain staining showed that starting on postoperative day 7, Propionibacterium acnes injected segments with endplates replaced by fibrovascular tissue. Progressive exacerbation was observed on postoperative day 14.	Immunohistochemical results showed TNF-α and CD3- immunopositive cells in the area of the endplates in the Propionibacterium acnes-injected segments at 14 days postoperatively.	-
Shan et al[24], 2017	At 3 and 6 months postoperatively, 7 of the 20 segments injected with Propionibacterium acnes were identified as having type II Modic changes, with no change in disc signal in the control group.	There were no significant differences in bone trabecular parameters between the groups.	-	HE and Safranin O and Fast Green staining showed endplate cracks in the Propionibacterium acnes -injected group, with no significant difference in the intervertebral disc tissue.	-	RT-qPCR results showed that the expression of TNF-α, IL−1β and IFN-γ mRNA was significantly up-regulated in rats injected with Propionibacterium acnes compared with those in the control group.
(4)Chemical-induced metabolic damage model						
Maruyama et al. [23],2024	-	In the model group, BV/TV, Tb.Th and BMD were significantly increased at 6w and 12w.	The total distance traveled and the number of feedings of the rats in the model group significantly decreased after the operation.	Safranin O and Fast Green staining showed reduced disc height, cracks and microfractures in the endplates and growth plates of the rats in the model group.	CGRP immunostaining showed that the percentage of CGRP-positive areas around the endplates of rats in the model group was significantly higher than that of the control group at 6w and 12w postoperatively.	-
(5)Lumbar instability model						
Liu et al[26], 2021	-	The endplates of mice in the LSI group were porous.	The time of voluntary activity of mice in the LSI group was significantly decreased compared. The frequency of hind paw retraction was significantly increased.	Safranin O and Fast Green staining showed degenerative changes and bone marrow cavities in the endplates of LSI mice.	Immunofluorescence staining showed CGRP-positive nerve fiber innervation in the endplates of LSI group mice at 4w and 8w after surgery.	The qRT-PCR results showed a significant increase in COX−2 mRNA, and PGES mRNA expression in the LSI group at 8w postoperatively compared with the sham-operated group.

*W* Weeks, *L* Lumbar, *Ca* caudal vertebrae, * EP* endplate, *MIA* monosodium iodoacetate, *ME* muscle embedment, *NPE * nucleus pulposus embedment

### Literature quality evaluation

In animal studies, all studies did not specify sample size calculations and inclusion and exclusion criteria, did not hide animal grouping schemes, and did not blind evaluate the results. Therefore, these items were not scored. Detailed information about literature quality evaluation is shown in Table 3.

**Table 3:** Quality evaluation results of animal experiments

Author	Random sequence generation	Reporting the reasons for excluding animals	Declaration of potential conflict of interest and funding
Han et al[20],2016	Y	Y	Y
Zuo R[25],2020	Y	N	Y
Morisako et al[27],2022	N	N	Y
Wang et al[28],2023	Y	N	Y
Lai et al[29],2023	Y	Y	Y
Chen et al[21],2016	N	Y	Y
Dudli et al[22],2016	Y	N	Y
Shan et al[24],2017	Y	N	Y
Maruyama et al[23],2024	N	Y	Y
Liu et al[26],2021	N	N	Y

*U* unclear, *N * no, *Y * yes

## Discussion

The pathogenesis of Modic changes mainly involves biomechanical loading, inflammatory response, low-toxicity bacterial infection and genetic factors. Among them, Propionibacterium acnes, as a key causative agent, can invade the anaerobic environment of the intervertebral disc through the blood circulation and induce Modic changes [30–35]. The results of this study showed that injection of Propionibacterium acnes into the intervertebral disc resulted in Modic type I or II changes of the endplates, which were accompanied by intervertebral stenosis, endplate cracks, and significant elevation of inflammatory factors, such as TNF-α, IL-1β, and IFN-γ [21, 22, 24]. It is worth noting that the pathological changes caused by this bacterium are different from those caused by Staphylococcus aureus (the latter mainly leads to intervertebral disc inflammation rather than endplate Modic changes) [21].

In addition to the infection model, other modeling methods accordingly revealed different mechanisms of induction. Monosodium iodoacetate (MIA) injection induces endplate defects, microfractures, and pain behavior in rats by inhibiting the glycolytic enzyme GAPDH. Where the 1 mg dose balances efficacy with safety based on successful induction modeling (increased lethality at higher doses) [23, 36–38]. In the autologous nucleus pulposus transplantation model based on the theory of “intravertebral disc rupture” [20, 39], the rat L5 vertebral endplates showed low signal in T1WI and medium-low signal in T2WI, accompanied by up-regulation of the expression of IL-4, IL-17, and IFN-γ, at 12–20 weeks after surgery [20]. The construction of this model usually requires higher surgical skills, takes more surgical time, and consequently incurs higher surgical costs. Injury or overstimulation of the paravertebral ligaments may lead to the formation of vertebral osteophytes during the specific operation of model preparation.

Mechanical damage modeling provides diverse research tools. Endplate degeneration with high TNF-α expression is triggered 30 days after caudal vertebral fracture in mice, but the extent of injury is difficult to quantify and MRI typing is lacking [25]. The caudal vertebra was chosen for the modeling process, as the surrounding tissues have less influence on the modeling process and can be positioned under direct vision. However, the small caudal vertebrae of mice may be difficult to manipulate, and the degree of damage caused is not easy to control and quantify. A model of lumbar instability constructed by resecting the supraspinous and interspinous ligaments of the L3-L5 vertebrae, µCT showed porous degeneration of the endplates in injured mice. However, MRI examination was also missing, failing to further define the type of injury [26]. The research has found that endplate injuries accompanied by vacuum sign are closely related to severe intervertebral disc degeneration and lower back pain [40, 41]. Morisako et al. [27] modeled endplate injury by creating a surgical incision in front of the fibrous ring based on this theory. The rats in the injury group showed pain-induced spontaneous behavioral changes, and the lumbar endplate region exhibited TIW1 and T2W1 low-signal changes on MRI. This model helps us to understand the mechanisms of pain and pathophysiology caused by endplate injuries. However, at the same time the model requires a high level of surgical manipulation, and controlling the severity of endplate injury in rats is a difficult task.

Wang et al. [28] and Lai et al. [29] constructed the rat Modic changes model by using terminal plate puncture combined with injection of inflammatory factors. The MRI showed Modic I-type changes with low signal at T1WI and high signal at T2WI in the injury area. The µCT and histologic findings showed disruption of the endplate structure. Behavioral tests revealed a significant decrease in pain threshold in rats. Furthermore, the research shows that the TNF-α injection group exhibited more significant Modic changes compared to the PBS group, but the pain behaviors were similar, suggesting that the injury of the endplate is the main cause of the pain rather than the type of injection. This model induces the phenotypic characteristics of clinical Modic changes, and the structure of the intervertebral discs in rats is similar to that in humans. It has low cost, is easy to breed, and is suitable for repeated experiments. Furthermore, the surgical procedure of puncturing the endplate is feasible. Under fluoroscopic guidance, it can effectively ensure the puncture depth and angle, and control the degree of damage. However, it is worth noting that the injection should be operated slowly to avoid oozing out of the injection solution and affecting the molding effect.

Comprehensively comparing the various models, the infection model has a clear mechanism and strong clinical relevance, but requires strict asepsis. The MIA model is easy to manipulate and induces pain behavior with a single injection, but lacks MRI verification of typing. Autoimmune models need to be weighed against surgical complexity. Endplate puncture combined with injection of inflammatory factors has both precision and clinical relevance. In contrast, the caudal vertebra fracture and lumbar instability models need to be refined for MRI typing validation (Table 4). In summary, different modeling methods have different advantages and disadvantages, and it is recommended that the model be preferably selected according to the research objectives and experimental budget. Also this study has some limitations. According to the STAIR checklist, the 10 included studies lacked specific evaluations for four items and the description of methodological details was inadequate, resulting in relatively low quality of the literature. Furthermore, review-type studies themselves have certain limitations. Although the search has covered the primary literature, there may still be deficiencies in incomplete database searches that do not cover gray literature and small language studies. Due to the different evaluation criteria in various literature, some data cannot be quantitatively combined or analyzed by subgroups, and there may be heterogeneity in the research data.

**Table 4:** Summary of Advantages and Disadvantages of Different Models

	Advantages	Disadvantages
Nucleus pulposus implantation model	Signal change in the endplate area	Difficulty of surgical operation
		Long operation time and increased risk of infection surgery
		Large damage to surrounding muscles and ligaments
Mechanical damage model	High clinical relevance	Lack of clear MRI typing in a model of caudal vertebral fracture
	Inflammatory factor injections have precision	
	Radiologically positioned and easy to operate	
Bacterial infection model	The mechanism of infection is clear	Strict aseptic operation
	High clinical relevance	Unharmonized standards for injectable doses
Chemical-induced metabolic damage model	Easy operation	Lack of clear MRI typing
Lumbar instability model	Consistent with the process of disc degeneration in humans	Lack of clear MRI typing
	The surgical program is mature and relatively simple to perform	More applications for low back pain models

## Conclusion

Animal models are an important vehicle to assist in the exploration of the mechanisms underlying disease. A good animal model usually needs to have features such as simple methods, high modeling rate and strong repeatability. With the widespread application of MRI technology in clinical practice, people have gradually paid attention to the relationship between endplate Modic changes, endplate injuries and low back pain. At the same time, the endplates, as the main pathway for nutrient supply to the intervertebral disc, are also closely associated with disc degeneration. Exploring the pathophysiological mechanisms of endplate lesions can help improve clinicians’ understanding of disease etiology, improve treatment efficacy, and even elevate preventive and therapeutic strategies to the molecular targeting level. The above model provides an important reference for exploring the mechanism of endplate lesions and the discovery of corresponding therapeutic strategies. However, the occurrence process of diseases in animal models is not completely equivalent to that in the human body, and there is currently no unified standard for clinical judgment of such models. Therefore, when choosing an animal model, multiple considerations should be integrated.

## Supplementary Information

Supplementary Material 1.

## Data Availability

All data generated or analysed during this study are included in this published article.

## Abbreviations

LBP: low back pain
PRISMA: Preferred Reporting Items for Systems Reviews and Meta-Analyses
MCs: Modic changes
BVN: Basivertebral Nerve
IBNRA: Intraosseous Basivertebral Nerve Radiofrequency Ablation
STAIR: Stroke Therapy Academic Industry Roundtable list
MIA: Monosodium Iodoacetate
ME: Muscle Embedment
NPE: Nucleus Pulposus Embedment
